# Exploring Stress and Coping in Caregivers of Children with Pulmonary Vein Stenosis: A Mixed-Method Study

**DOI:** 10.3390/children11081008

**Published:** 2024-08-17

**Authors:** Mark Fuller, Christina Ireland, Rachel Zmora, Kathy Jenkins

**Affiliations:** 1 Department of Cardiology, Boston Children’s Hospital, Boston, MA 02115, USA; 2 Department of Preventive Medicine, Northwestern University Feinberg School of Medicine, Chicago, IL 60611, USA; rachel.zmora@northwestern.edu; 3 Department of Pediatrics, Harvard Medical School, Boston Children’s Hospital, Boston, MA 02115, USA

**Keywords:** stress, coping, pulmonary vein stenosis, mixed methods

## Abstract

(1) Background: This mixed-method study aims to identify and describe factors associated with acute and long-term parenting stressors and coping strategies in caregivers of children with intraluminal pulmonary vein stenosis (PVS). (2) Methods: Parents of children with severe PVS were recruited from a large quaternary hospital to complete a survey that included demographics, the Pediatric Inventory for Parents (PIP), and the Coping Health Inventory for Parents (CHIP). We determined the Social Vulnerability Index (SVI) based on self-reported home address. A subset of caregivers completed a 60 min semi-structured interview via Zoom exploring the impact their child’s diagnosis had on their family; experience managing stress in the hospital and at home; current resources and processes for coping; and potential recommendations for hospitals to build resilience and coping. We used multivariable linear regression to examine the association between SVI and parental stress and coping while adjusting for possible confounders. Thematic analysis identified themes related to stress and coping. Finally, we assessed instances of convergence and difference between the qualitative and quantitative results. (3) Results: Participants included 32 caregivers who were 91% female with a mean age of 39 years. The children of participants were 66% female, with a mean age of five years. The parents reported a high amount of stress with an average PIP score of 120, nearly 46 points higher than similar studies in the congenital heart community. We observed no significant associations between SVI and either parental stress or coping in adjusted models. We identified 13 themes, including medical care, hospital, family, support systems, and home medical routine or support. (4) Conclusions: Our study found high levels of illness-related parental stress among caregivers of children with PVS. Stress evolved over time from what caregivers described as ‘survival mode’ to a future-oriented outlook. Currently, caregivers rely heavily on support networks that are not available to all caregivers or may experience strain over time. Caregivers indicated that communication and parental role functioning were coping strategies that could be better supported by providers and health systems.

## 1. Introduction

Pulmonary vein stenosis (PVS) is a rare pediatric disorder that involves recurrent intraluminal obstruction of the pulmonary veins and can lead to pulmonary vein atresia, pulmonary hypertension, right heart failure, and death [1]. Roughly 1.7 in 100,000 children under the age of two are reported to have PVS [2]. PVS can occur in infants independently, in those with prematurity, chronic lung disease, and, more commonly, in conjunction with complex congenital heart disease. Managing this disease is unlike other cardiac defects because reoccurrence of the stenosis is expected [3]. Historically, PVS has a poor prognosis, with mortality rates as high as 60% by the age of two years, though outcomes are improving with new multi-modal approaches [1,4].

Current research on PVS has focused on treatment and management; however, the impact of PVS on families is not well understood. Each patient and family brings a unique set of circumstances, and no two patients are the same [3]. While many centers offer state-of-the-art multimodality treatment, the provision of patient- and family-centered care requires much more than medical therapy and intervention [5]. 

Parents with medically ill children experience higher levels of parenting stress as a result of their child’s illness compared to parents of healthy children, with some exhibiting acute signs of post-traumatic stress disorder (PTSD) [6]. Illness-related parental stress can be defined as parenting stress that is associated with raising a child with a chronic illness [7]. The combination of illness-related stressors in conjunction with typical stressors associated with raising a child necessitates examining illness-related parental stress as a dimension of the broader parental stress experience.

This study identified and described correlates of acute and long-term parenting stressors and the process of coping in caregivers of children with PVS. The findings provide insight to healthcare providers when designing and implementing support systems and resources to mitigate stressors and promote resiliency. No literature currently exists examining parental stress in parents of children with PVS, creating a substantial gap in our understanding of illness-related parenting stress in this population.

## 2. Materials and Methods

We used a mixed-methods convergent parallel design. Quantitative and qualitative data were collected concurrently and then compared for areas of convergence and difference.

### 2.1. Inclusion Criteria

Participants were screened for eligibility by a PVS Nurse Practitioner (NP) who works with the cohort of PVS families in this study. To be eligible to participate, participants had to be a parent or guardian of a child with intraluminal PVS involving at least 2 veins and received at least one dose of Imatinib Mesylate (Gleevec), indicating severe PVS. All children obtained PVS-related care between July 2010 and July 2022 at Boston Children’s Hospital (BCH). Additionally, participants had to speak, read, and understand English and have access to an internet-enabled device. 

### 2.2. Recruitment and Enrollment

A convenience sample of caregivers of children with PVS was recruited from cardiology clinics and the internal PVS database by the NP via a recruitment email. A total of 57 potential participants were identified. An initial recruitment email was sent by the NP via secure email informing participants of the purpose of the study, eligibility criteria, and participation information. After participants expressed interest, the first author obtained informed consent using REDCap. Following enrollment, participants were sent a secured REDCap survey via email to complete. At the end of the survey, participants could indicate if they were willing to participate in an interview via Zoom.

For quantitative data, we estimated a sample of 32 participants would provide 80% power to detect the impact of the severity of PVS diagnosis on parenting stressors and coping strategies based on the available literature for congenital heart disease (CHD) [8]. For qualitative data, we planned to interview 10 participants by convenience sample or until thematic saturation was achieved. 

### 2.3. Qualitative Interviewer

The interviewer (first author) is a cisgender gay male (he/him) who worked within the cardiology program at the hospital as a music therapist at the time of the study. He was a Board-Certified Music Therapist completing their Master of Public Health degree internship. When conducting interviews, the facilitator provided participants with a background on their role as both research investigator and music therapist within cardiology, informing them of their interest in this research topic due to their clinical work and in fulfillment of their degree before beginning the interview. 

### 2.4. Data Collection

#### 2.4.1. Quantitative Data

Participants were administered an online survey via REDCap that assessed demographic and contextual data, as well as parental stress and coping. The demographic survey captured information on the caregiver and patient’s age, gender, race, ethnicity, education, and socioeconomic status. 

The patient’s disease severity and medical and hospitalization history were obtained from their medical record by the NP. Newly diagnosed PVS was defined as being diagnosed within six months of enrollment. Recurrent PVS was defined as needing ongoing cardiac catheterization to address active disease. Stable PVS was defined as not needing cardiac catheterization to address active disease for at least six months, followed by the withdrawal of the use of Gleevec medication without a change in disease state.

Using participants’ addresses, we determined their Social Vulnerability Index (SVI) score. The SVI measures resources a caregiver may have in their home environment to respond to an external stressor. Using U.S. Census data, it rates the social vulnerability of a community from 0.0 to 1.0 by ranking 16 social factors (e.g., poverty, lack of vehicle access, and crowded housing) into four themes (socioeconomic status, household characteristics, racial/ethnic minority status, housing type/transportation) to produce their overall vulnerability [9]. A higher SVI value indicates greater social vulnerability. 

We assessed parental stress using the Pediatric Inventory for Parents (PIP). The PIP is a validated tool used to assess parental stress in parents of chronically ill children [7]. The 42-item instrument has four domains: communication, emotional distress, medical care, and function. The domains are measured on two scales: frequency and difficulty. The participant ranked frequency by noting ‘How Often’ (e.g., 1 = never, 2 = rarely, 3 = sometimes, 4 = often, and 5 = very often) the event occurred in the past week. The participant ranked difficulty by noting ‘How Difficult’ (e.g., 1 = not at all, 2 = a little, 3 = somewhat, 4 = very much, and 5 = extremely) the event was in the past week. Each domain is totaled separately for a sum score of frequency and difficulty. The frequency and difficulty scores for each domain were then combined to yield an overall frequency and difficulty score ranging from 42 to 210. A higher score indicates a greater frequency and difficulty in stress.

We assessed parental coping with the Coping Health Inventory for Parents (CHIP). The CHIP evaluated parental coping and the perception of managing family life when caring for a child who has a serious and/or chronic illness [10]. The 45-item instrument has three subscales: Coping Pattern I: Family integration, cooperation, and an optimistic definition of the situation; Coping Pattern II: maintaining social support, self-esteem, and psychological stability; Coping Pattern III: understanding the healthcare situation through communication with other parents and consultation with the health care team. The items were rated by the helpfulness of the coping behavior from 0 to 3 (e.g., 0 = not helpful; 1 = minimally helpful; 2 = moderately helpful; 3 = extremely helpful). Subscale and total scores were calculated by summing individual items.

#### 2.4.2. Qualitative Data

We conducted ten in-depth interviews (IDI) from September 2021 to August 2022 to explore the experience of parental stress and coping in caregivers of children with PVS. Interviews were 60 min in duration and conducted via Zoom by the first author. The interviewer utilized a semi-structured interview guide, which included five questions covering the caregiver’s experience receiving their child’s diagnosis, stressors associated with diagnosis and treatment, and coping strategies (Supplemental Table S1). The interviewer utilized probing questions to obtain clarity on a caregiver’s responses. The first author recorded and transcribed the interviews and disseminated them to the analysis team (R.Z., C.I.). 

The IDI guide was developed by the first author based on relevant literature on stress and coping in the pediatric heart disease community and revised in collaboration with the other authors. The guide was reviewed by a caregiver from the PVS community who was not eligible for the study to ensure the questions were relevant and appropriate. 

### 2.5. Analysis

#### 2.5.1. Quantitative Data

Prior to analysis, data were reviewed for accuracy and completion. Demographic data for parents and children were summarized using descriptive statistics as appropriate: frequencies, interquartile range, and percent for categorical and dichotomous variables; means and standard deviations and minimum and maximum scores for continuous and ordinal data. 

We used multivariable linear regression to assess the associations between our primary exposure, SVI, and the outcomes, PIP and CHIP while accounting for confounding by potential covariates. The models were adjusted for parent age, sex (male vs. female), race (defined as White vs. other), marital status (defined as partnered or in a relationship vs. single), and the number of children they were caring for, as well as the child’s age and illness severity (newly diagnosed or recurrent vs. stable). Quantitative analyses were conducted in R V 1.3 [11]. We used *p* < 0.05 to determine statistical significance for all analyses. 

#### 2.5.2. Qualitative Data

Individual interviews were organized in NVivo 12, and data were analyzed using Braun and Clark’s six phases of thematic analysis [12,13]. The six phases of thematic analysis include familiarization, coding, generating themes, reviewing themes, defining and naming themes, and writing up. The analysis team (MF, CI, RZ) utilized open coding to generate themes. Disagreement was resolved through consensus. After the preliminary analysis was completed, member checking was performed with a subset of interviewees to ensure that the themes accurately represented the ideas of the participants.

#### 2.5.3. Mixed Method

The resulting themes were then compared against the quantitative results to identify any combination of convergence and/or difference between the data sets. NVivo was utilized to query connections between themes and compare themes to our quantitative results. 

## 3. Results

### 3.1. Participant Characteristics

Our sample consisted of 32 caregivers who were 91% female with a mean age of 39 years. Most caregivers identified as White (81%), with the remaining caregivers identifying as Asian (3%), Black or African American (6%), Hispanic (3%), Black or African American and Native American (3%), and one respondent who preferred not to answer (3%). Caregivers were highly educated, with 41% having completed graduate education. A total of 41% of caregivers were working full-time, 28% were working part-time, and 31% were unemployed or not working during the time of the study. Most caregivers (91%) were married or in a marriage-like relationship during the study period. The median SVI for our sample was 0.4 (IQR = 0.5), which corresponds to residing in a community with low social vulnerability (Table 1).

The children of participants were 66% female, with a mean age of five years old. The majority of children had either stable PVS (56%) or recurrent PVS (41%), with a small portion of children who were newly diagnosed with PVS (3%). Children traveled a median distance of 800 miles to receive PVS care. On average, children were hospitalized three times in the past year with a median admission length of two days. However, there was considerable variation, with children experiencing between 0 and 10 admissions with admission lengths ranging from 0 to 365 days. 

### 3.2. Parenting Stress, PIP Survey

Caregivers reported an overall average PIP score of 120.3 (SD = 35.6) with an average difficulty score of 116.8 (SD = 32.5) (Supplemental Table S2). We used multivariable linear regression to assess the association between SVI and the four domains of parenting stress: communication, emotional distress, medical care, and role function (Table 2). We observed no association between SVI and any domain of parenting stress. Communication-related stress was higher among parents of children with recurrent or newly diagnosed PVS (B = 5.8, SE = 3.5, *p* = 0.11); however, this association was not statistically significant. We found that the number of children (B = 1.9, SE = 1.0, *p* = 0.08) and patient age were positively associated with role functioning-related stress in caregivers (B = −0.8, SE = 0.5, *p* = 0.12); however, these associations were not statistically significant. Finally, female caregivers had higher emotional distress (B = 14.6, SE = 9.3, *p* = 0.13); however, these associations were also not statistically significant. 

### 3.3. Coping Behaviors, CHIP Survey

Caregivers reported average CHIP scores of 37.2 (out of 54), 15.6 (out of 24), and 25.8 (out of 48) for the coping patterns of social support, communication, and family cohesion, respectively (Supplemental Table S3). We used multivariable linear regression to model the association between SVI and CHIP subscales adjusting for covariates (Table 3) and found no statistically significant associations. Caregivers with a higher SVI tended to have higher communication-related coping behaviors (B = 3.6, SE = 2.5, *p* = 0.17). Caregivers with a higher number of children tended to report lower family cohesion-related coping behaviors (B = −1.4, SE = 0.9, *p* = 0.13). 

### 3.4. Qualitative Themes from Caregiver’s Interviews

From our in-depth interviews, three domains of stressors and coping strategies emerged with caregivers: medical diagnosis and hospitalization, family unit and lifestyle, and mental health and coping (Table 4). For the purposes of this paper, we highlighted some of the prominent themes within the domains of medical diagnosis and hospitalization, family unit, and mental health and coping.

#### 3.4.1. Medical Diagnosis and Hospitalization

Caregivers described stress associated with both medical care and the hospital environment. Key among these experiences were receiving their child’s diagnosis, transitioning to their treatment hospital, and lengthy hospitalizations. Coping strategies associated with these experiences included accessing a PVS Nurse Practitioner (NP), gaining medical knowledge and proficiency in PVS-related terminology, and becoming familiar with the treatment journey for their child’s disease.

*Diagnosis.* Receiving a child’s diagnosis of PVS was a key stressor for caregivers because of the severity and uncertainty of the prognosis. After receiving a PVS diagnosis, parents were faced with the possibility of their child dying. Several caregivers shared that they were offered palliative care prior to initiating treatment for PVS. Some hospitals lack the resources or expertise to treat PVS, resulting in parents seeking out alternatives on behalf of their child. One parent described this process:

“I am pretty sure they were just going to send us home on hospice. They didn’t really say that, but I think um, they didn’t give us a plan. […] So, we asked if we could get other opinions, and it was interesting, they were like well yeah, we can do that we can send your stuff over to Texas and Boston. And we were like, okay? Why do we have to drag our feet, and why did we have to ask. So, I don’t know.”

Caregivers reported being frustrated and overwhelmed by having to identify treatment options for their child, which they felt should have been the responsibility of their local hospital. Many parents expressed a need for local facilities to improve education and knowledge on PVS among clinical staff to support timely and efficient referral to facilities with treatment options. In the absence of needed support during this process, caregivers felt isolated and alone. One caregiver described their experience as follows:

“We are sitting here, nobody knows what to do, they are addressing like if she is safe right now, is she okay right now, like ICU stuff. But you can tell no one, there, we felt like we were being avoided.”

*Lengthy hospitalization stays*. Caregivers noted the toll that lengthy hospitalizations take on both their physical and emotional well-being. Two key stressors were the lack of continuity of care they received and the constant change in medical fragility that their child endured. 

First, the lack of continuity brought uncertainty in understanding their child’s treatment team early on in diagnosis. This uncertainty was noted to cause anxiety and stress related to the quality of care that their child was provided. Caregivers noted the importance of a provider knowing their child’s behaviors and symptoms to assess and evaluate care options. One caregiver noted stress occurred when,

“[…] anytime that the team changes the anticipation of a new attending, the anticipation of like a weekend or a holiday, or knowing that the people that know [patient name] are not going to be in the hospital […]”

However, it was noted that roles such as the PVS NP, which remain constant throughout the hospitalization, are critical to the trust and safety of a caregiver’s well-being. This allowed the caregiver to feel that the quality of their child’s care was taken care of.

Having a provider like an NP that remains constant for families allows them to build rapport with their provider. This is evident in how caregivers access their PVS NP for medical advice, emotional support, and familial decisions beyond their PVS child’s medical needs. Specifically, non-medical support was described as navigating housing, transportation, financial challenges, and sibling caretaking. Caregivers note that their relationship with their NP is holistic and family-centered. They also mention that access to their provider is important both when they are at the hospital or home. A caregiver shares,

“I take advantage of Christina in the hospital but mostly out of the hospital too. […] I have her on text and that’s incredibly helpful to us. Even if I have a little question or I’m like, kind of worried about something and I have her, I have her when I’m sitting right here. I have her when I’m sitting on my couch.”

Second, lengthy hospitalizations involved several transitions in the level of medical attention a PVS child needed. This heightened caregiver stress due to the constant change in medical fragility that their child was in and the rapidly changing severity of their diagnosis. Caregivers noted the high number of transitions between acute and intensive care, in addition to the number of medical procedures that occur in a hospitalization, which are difficult to cope with. With this, caregivers reported the importance of the PVS NP in guiding them through the treatment journey and helping them understand the importance and necessity of the rapid shifts in medical care. One caregiver described her experience as,

“[…] we just came back through the emergency department after talking to Christina um and then we were whisked away into the ICU. […] Christina became very, very active because at that point we understood it was PVS, right, and I would say that if not for Christina I wouldn’t have the understanding of one, what the impact is today, but two what the plan is and what the progress has been. She has been instrumental in kind of being our north star in this.”

*Transition to Treatment Hospital.* Caregivers describe the transition from their local hospital to their PVS treatment facility as causing stress due to a lack of awareness of their child’s disease and treatment course in the transition. Many local hospitals provided only supportive care. For most of the caregivers interviewed, treatment began with the transfer of care to a quaternary care center. One caregiver described the frustration with the lack of local treatment options,

“It is never having me going to Boston Children’s that is giving me the anxiety, it is the travel because we fly. A lot of equipment. We love Boston Children’s, I don’t understand why it is like, frustrating where we live, … we live near those big teaching facilities, and so it is very frustrating that we cannot receive our care locally.”

Caregivers who traveled a significant distance for care articulated being upset, overwhelmed, and tired from the lack of medical care available closer to home. Several caregivers specifically mentioned leaning on the PVS Nurse Practitioner at this time. Traveling for care resulted in added stressors, including identifying housing, transporting a medically fragile child, and affording associated expenses.

Caregivers also share that the transition to their PVS hospital caused stress in understanding their child’s diagnosis. They mention that their local hospital often was unable to describe the diagnosis and treatment options for PVS and relied on the transfer to their PVS treatment hospital to relay information to caregivers. This led to caregivers needing to access online resources independently to attain information on their child’s disease. Many reported accessing online sources like Google to receive information. One notes,

“It has been a steep like learning curve as far as like understanding just some of like the general like anatomy components of PVS, and then along with like what it means for him and what like why it’s a problem and all the different things that it affects as far as like his clinical signs go, and like how to tell if it’s getting worse or like what to look for and like, but like all the things like it was just a lot of learning and a lot of trying to like understand all these like different acronyms.”

Caregivers further acknowledge their interest in learning medical and anatomical terminology given to them so they can better participate and engage in their child’s treatment. However, several of them note that prior to their child’s diagnosis, they were not familiar with medical terminology. This also carried stress as they began to identify online materials to learn about PVS due to the limited information that often led to severe outcomes, like death. Caregivers outline the difficulty in attaining accurate and comprehensive information on their child’s disease to assist them in learning how to care for and advocate for their child’s rare disease. Relying on online resources appears to be an independent method to prepare for discussions with their medical team and to understand the information provided to them. Additionally, caregivers report a desire to learn about their child’s disease from their providers and to have information accessible for conversations with their team.

#### 3.4.2. Family Unit and Lifestyle

The family unit is deeply affected by having a child with PVS. Even at home, away from the hospital environment, PVS impacts everyday life. Caregivers described significant stress from meeting the home health needs of the child with PVS, balancing the needs of siblings, and meeting financial obligations. 

*Medical needs.* For many families, schedules are dictated by the medical needs of the child with PVS. Caregivers’ days at home were focused on providing medical attention and care to their children with PVS. Furthermore, parents are responsible for organizing therapies for their children, which may occur multiple times a week or day. Caregivers often shared that they had minimal time for breaks or to spend with their partner or other children, which resulted in feelings of exhaustion and burnout. They also noted that the amount of medical knowledge needed to monitor symptoms and administer medications greatly limited the individuals who could perform these tasks. Caregivers shared that the medical demands of their child required close attention and skills that were difficult for a layperson to perform.

“So, she, you know maybe up to about a year old so six months ago we had to feed her kind of you know every, every three hours and it’s not you know taking a bottle down in 15 min. It’s using the G tube, the pump, and that things running over an hour and a half and so was a year of, someone’s up, you know turning that thing on, turning that thing off through all hours of the day.”

Caregivers in a relationship who lacked support systems shared the challenges of balancing caring for their PVS child with one another. They often described one caregiver taking on the majority of the responsibility to care for their child with PVS.

“Like my relationship with my husband had been strained for a little bit, because it was like he was afraid to take care of her for a while, and I would get frustrated because then it meant that I really had no break. But he got better now, um, so that was an issue for a little bit. But then I tried to give him and myself some grace that we are taking care of a- we are doing nursing tasks, and we have zero formal education. But it has definitely gotten better.”

However, caregivers with positive support systems were able to train close relatives, such as their parents, to care for their child with PVS. When this was noted, these caregivers often described the positive impact of being able to take a break and care for themselves.

“Yeah, so we have, my mom and siblings, and my in-laws, my mother in-law, and my husband’s siblings have all been our main team. They are the ones over time we have trained them to care for [patient name]. They are our only babysitters basically. We can leave the kids with. That is a little stressful because we have not gotten a babysitter. There has not been a comfortable like, ok we are comfortable with them. And it is interesting even like now as normal as she is, you still have thought of I need someone to know her well enough to call me and say something is not right. Or you know something is weird with her breathing, or she is acting different than she normally does. So we have pretty much stuck with like our family for most of our community.”

The burden placed on the families by PVS continues beyond the initial years of treatments. One caregiver of an adolescent child noted,

“…school started here two or three weeks ago so all the schedules got flipped. Whether it’s dance, gymnastics, and soccer. And then all the speech therapy, OT, piano, all of it shifted. And so, now it’s also okay, it’s um, it’s harder because with a kiddo with PVS, even a healthy kiddo with PVS, you’ve got three more therapies a week. And you got all these other complicated things that you are working through. And so now, I am grinding my teeth at night, and um, not sleeping well, um, and I know that while it is stressful for most parents with back to school, there is a lot more irons in the fire with a kiddo with PVS. So…”

Even during periods of stable disease, families must schedule around the medical needs of the child with PVS. As the child ages, caregivers are sharing their challenge in organizing schedules among their family to create space for their child with PVS to engage in community events unrelated to their medical and therapeutic care. While this creates stress, caregivers note positive emotions related to this challenge, such as hopefulness and happiness, when they see their child engaging in activities with peers their age.

Another stressor on the family unit was their need for health insurance to maintain access to their child’s PVS treatment. Half of the caregivers discussed maintaining health insurance in their interviews. Caregivers noted being highly aware of their need to maintain adequate healthcare coverage to afford treatment for their child. In addition to health insurance, caregivers valued jobs with flexible or work-from-home schedules. Several caregivers described transitioning from dual to single-income status due to the significant time needs of caring for a child with PVS. 

*Siblings.* Many caregivers reported difficulty balancing caring for their child with PVS with their sibling or siblings at home. This task was made more difficult because many caregivers had to travel significant distances to obtain care for their child, leaving the siblings behind in the care of another parent or relative. One parent described the thought process of choosing when to leave the hospital to spend time with their children at home,

“And then I think the other part of it that really affects it for us is having [sibling name] at home and like trying like, I don’t want to leave [patient name] on the good days ‘cause I want to be around for the good days, but I also know I‘m never going to leave on a bad day, so it’s like really trying to balance that. Are we in the right headspace to be good parents […]?”

The difficulty balancing hospital and home life is in part due to the precarious nature of their child’s health. Parents expressed intense fear of their child’s medical status changing when they were not present at the bedside. Even away from the hospital, this fear impacted their parental functioning when at home with their other children.

Parents described the importance of engaging in activities as a family unit to create positive memories. Taking vacations, seeing family, or having play dates with peers and other children were commonly discussed among caregivers. However, these caregivers typically were those of children with stable PVS who were out of the hospital and had a future-oriented mindset. 

When siblings were older, parents faced the challenge of explaining to the siblings what PVS is and why it necessitated an altered lifestyle, including frequent medical appointments and hospitalizations. A caregiver stated,

“[…] It is extremely hard to explain to your daughter and she is like, well nothing is wrong with my heart right? And you know [child’s name] gets to miss school because she has to go to all these different doctor’s, and she is like well why does she get to miss school. […] So, it is hard to explain to her all that, but it is also hard to explain to the other child like oh you may die one day. It is extremely stressful to live a normal life with your kids, there is jealousy, there is jealousy in every family. But this is like way more than that, way more complex, so that has been hard.”

The impact on siblings communicates various challenges for caregivers due to the lack of resources currently available to support the emotional well-being of a sibling through the hospitalization and treatment journey. Caregivers noted the importance of family-centered care from clinical disciplines like child life specialists and music therapists that provide supportive spaces for siblings to process their emotions and develop coping strategies in both hospital and at-home settings. 

*Finances.* Families often experience financial hardships while caring for a child with PVS. One parent is often forced to go on leave from their job or quit working to be with their child in the hospital or provide medical care for their child at home. Notably, this strain was reported by previously financially stable families. One caregiver who expressed being financially stable shared,

“I didn’t go back to work for two years. Um, she was just too sick and I needed to be home for her and be her caregiver. Which is totally fine. That is like in my mind, a blessing that I could do that. I don’t take that for granted, but it certainly shifted, you know, there is no income for me. And we are spending more money anyways. So it was kind of like, okay? And you are in survival mode for a while that you just buy food and buy what you need, and you can’t even think about budget because you are just surviving this time…”.

Caregivers shared that job stability was a significant stressor as they were dependent on access to healthcare and financial resources to access their treatment and care for their child. Budgets were further stretched by the need for housing and transportation to access their treatment facility.

“[…] the stress of my husband keeping his job just for the health insurance. We didn’t really have that stress before, we just always used common sense.”

For some caregivers, they reported having to leave their full-time work to care for their child’s health. This left them with financial stress and unexpected expenses to support their child’s medical needs. The financial stress caregivers noted led them to reduce their spending, which increased their isolation and ability to maintain their desired lifestyle.

#### 3.4.3. Mental Health and Coping

The mental health of a caregiver of a child with PVS is affected by balancing employment responsibilities, sleep deprivation, and medical trauma from their child’s hospitalizations. Coping strategies evolve from a state of survival to meet their basic needs towards a future-oriented mindset for lifestyle supports like therapy, exercising, and being outside. Support systems often involve a partner who is experiencing the process alongside the primary caregiver and extended family and friends, like parents, who assist in various tasks like babysitting, providing dinners, or supporting financial aspects of their life.

*Mental health.* Caring for a child with PVS impacted the mental health of caregivers, who frequently reported symptoms of anxiety, depression, and burnout. Parents who remained in the workforce were solely responsible for the family income and access to health care, while parents caring for a child with PVS at home were faced with meeting the needs of a medically complex child. One parent described the experience of becoming a full-time caregiver for their child:

“Um, for me I stay at home with the kids. And he works full-time and another part-time job because I am not working. For me being at home was all for doing meds and feeds and all that kind of stuff, was completely incapacitating. Um, I could not take care of myself. Um, it is very emotional [begins to cry], very heavy. Probably the lowest of my life.”

The strain on caregivers to fulfill all their child’s medical needs while also creating space for play and bonding frequently impacted caregivers’ ability to get sufficient sleep. Sleep deprivation was commonly experienced across all points of the treatment journey, both within the hospital environment and while at home. Sleep deprivation frequently worsens symptoms of anxiety, depression, and burnout experienced by caregivers. Caregivers reported interest and need for mental health care for themselves. For some caregivers, this was a new need, and for others, caring for a child with PVS increased their need for mental health support in the form of psychotherapy or medication. However, despite recognizing the importance of taking care of their mental health, caregivers often face barriers to accessing mental health care. These barriers included affordability, availability, and time to engage with mental health resources. One caregiver shared,

“I saw a psychiatrist before I had [patient and patient sibling name], but more so after I had them, um to get on medication. Because I was just so tearful, I would cry at the drop of a dime. Um, even now when like, we go to the doctor, to the hospital, I am just in shambles because of everything.”

Caregivers who experienced extreme mental distress often attributed it to the inability to access timely mental health care. They shared experiences of medical trauma associated with receiving their child’s diagnosis and processed their experience using analogies to describe feeling overwhelmed, anxious, confused, or in denial of their child’s diagnosis and treatment. One parent shared an analogy of a vacation gone wrong, stating,

“OK so you’re geared up for this trip you’re going to California right? Sunny skies, it’s gonna be beautiful, you might catch some waves, go out and get some dinner, maybe you went to Napa, I don’t know, and you get on the plane and you’re like this is awesome like I’m ready for California. And you know, you land, get out, walk off the tarmac and imagine you’re in Antarctica. That’s our journey, right? Like from expecting to have a healthy child and you’re ready to take that trip of a lifetime and you get out and you’re in somewhere else that you didn’t even expect you were going to be. That’s the analogy that I tell people.”

Mental health challenges continue for caregivers through treatment as their child may go through periods of stability punctuated by recurrent disease, which can happen at any time. The unpredictability of PVS was frequently cited as a stressor by caregivers. One caregiver described the experience as a rollercoaster,

“So, for me I think the moments that are most difficult are hearing the bad news. And you know I’m sure that you’ve gathered this from other folks that you’ve spoken with, but it’s, there’s a roller coaster ride to this thing, right? And so, you’re getting good, we’re getting good news at many moments in time and then there’s like oh two steps forward three steps backward, oh it’s the oh it’s back, and so those are the moments where it’s like just deflating.”

As a child’s disease stabilizes, the perception of stress related to the child’s diagnosis evolves. Caregivers report a shift from survival to a future-oriented outlook to care for their children,

“But PVS, it’s like cancer of the heart. It keeps coming back and keeps coming back. And um, that’s just in the acute phase, um where you are fighting for your child’s life. And for us that was a hard two years. Um, the third year we were starting to exhale a bit, and since then its yeah, mental health has been more about coping and finding our ways to, I would say, breathe every day. Um, but to gain a sustainable lifestyle.”

Caregivers share their inability to meet their basic needs and articulate a survival mindset (i.e., sleeping, eating, personal hygiene, and breaks from the bedside) when discussing coping in the initial phases of treatment. A shift towards a future-oriented outlook is seen as the child stabilizes and the caregiver seeks developmental supports and therapies, explores school and daycare needs, and identifies managing home life and routines.

*Self-care.* Caregivers frequently described being in ‘survival mode’ during the initial phase of diagnosis and treatment. Due to the overwhelming stress of this period, they focused on meeting the basic needs of themself, their child with PVS, and their immediate family. They described an inability to access coping mechanisms at this stage. One caregiver shared,

“I make sure that I shower every day. It’s the simple things. Take a shower, turn your music on, wash off the day, reset. That even when we were in the hospital, I was like I must shower because once I get into the cycle of not showering, that depression is a snowball effect.”

Finding a routine to meet their basic needs while at the hospital is difficult to create and maintain, which they explain can escalate their mental health challenges. This continues in the home environment with the demands placed on the caregiver and the lack of respite that they receive. Caregivers reported a lack of sleep and feeling sleep-deprived when unable to have periodic respite,

“I was very interested in if there was a home nurse that could come and stay while we were to grab dinner or something. But we didn’t qualify because [patient] was on a g-tube and oxygen and they felt that was I don’t know what the correct terminology is, where she was not easy enough to qualify, like with a trach or something more extensive and she would qualify for it, and I was like in my mind of course I understand that, but selfishly I was like I would like a break too.”

Caregivers noted the need to take breaks and care for themselves but often did not qualify for adequate care that could allow them to take breaks. They noted the importance of breaks of as little as an hour to be able to care for their well-being, but not being able to receive this. However, for the limited number of individuals who accessed respite care, they described the positive impact this had on their mental health.

“We have a home nurse that comes and helps us with [patient name]. And she does respite care, and she is also a great third set of eyes on her breathing, and all of her stuff, but um, so she takes that over, but also I can release that which is great. So, I am not the only one carrying that from day to day. And that is hard to have a medically fragile kiddo, and so to have someone who you can hand that over and release being a nurse and just be a mom. So, um, that is a huge stress relief.”

Those who did not qualify for home nursing often noted their skill in being able to care for their child but wanted a trusted individual who could provide them space for their own basic needs.

“We never qualified for nursing care in the beginning because she only had a g tube. And I kept saying like I am a nurse, I can do these things, I don’t really need somebody. I really just needed someone who could like hold her while I sleep.”

Caregivers discussed the importance of another trusted individual for their ability to step away from their parental role responsibilities and engage in activities like sleep, exercising, socializing with friends, and date nights with their partner.

*Support system*. Among our sample of caregivers, support systems extended beyond their immediate family members to include friends, faith groups, and support groups. These systems looked different for each family. Support systems filled many needs, including childcare, meals, and emotional and financial support (i.e., gifts, gift cards, money, etc.). 

With that, the complexity of care needed for the PVS child makes it difficult for caregivers to identify a babysitter who is affordable and equipped with the skills and training to take care of their child. Caregivers note that family and close friends often are the only people they can access for babysitting. They share the importance of having family trained to care for their PVS child so that they can take respite,

“It’s really cool from a, from a family unit perspective is like we, we have G tubes, we have medications, we have all of these things, an ostomy right? Like all of these pretty technical things that we have to learn and everybody in the family, not just me and my wife, grandma, grandpa, sister, everybody knows how to do these things for the most part. And so, where I think and [wife’s name] and I are very, we’re very fortunate to have that at home.”

Caregivers who reported not having a support system available to them who were trained to take care of the PVS child often expressed the stress of being isolated and unable to take breaks for their own needs due to the full-time care their child requires.

“You have to have a good support system with your family. And if you don’t, you struggle because, like for us you know, our parents aren’t comfortable with the meds and the g-tube, and the chemo and things, and oxygen. So, we have been restricted to only like, if we do get a chance, we will have a sitter for a few hours, and it’s not like you can’t take those two-day breaks where you can decompress and unwind…”

Some caregivers expressed frustration with family and friends when discussing their child’s health. Although caregivers acknowledged the complexity of their child’s health needs, they were frustrated by the need to repeatedly explain their child’s diagnosis. One caregiver said,

“I do try to talk to people, but I try not to do too much because then they ask a lot of questions and I have to explain and teach a lot. And that is almost harder and taxing and stressful for me. And no one in my family has done their own research to find out what is happening and what is really going on, and that would be really helpful if other people had been like I have taken the time to research and know what is going on.”

The need to provide repeated explanations to their support networks highlights the unique challenge of caring for a child with a rare disease. The rarity of PVS meant few members of a caregiver’s support system were aware of the disease, and therefore, they did not understand the severity of the diagnosis or the demands of its treatment, causing difficulty relating and empathizing with the caregiver’s experience.

To fill this gap, several caregivers articulated the importance of support groups both in the hospital and at home as they navigated caring for their child with PVS. They described the importance of shared experiences and building community among caregivers. Caregivers described the nuances that the PVS journey encounters, which makes it difficult to relate and connect with families of other diseases. They noted that there is a clear need to have a space with other families who are going through a similar journey to be able to bond and connect with them. One caregiver shared,

“I think the other part, is that now we are up in the cardiac unit and meeting people, you are in it for the long run, but up in the NICU you meet people with much more solvable problems. Not necessarily good problems, I will never say any of their journeys are easier, but there was a clear and final step.”

Another shared accessibility is key to creating the PVS community as these families are dispersed across the globe and not in high concentration. Due to this, it is difficult to find families within their local communities. The use of online platforms as a means for building community and seeking support has aided the growth of the PVS community. One caregiver shared,

“There is a PVS group on Facebook that I am still on. I mean that is good. There are not many people around you. I mean caring for a child with PVS is isolating so you won’t really have many people around you. So coping was me, I mean it was not a specific time, but you just go on the group and ask specific questions or just vent, so that was helpful.”

The importance of a PVS community is communicated across our caregivers as a necessity for building a space for connection, education, and support among those who are going through similar experiences. We saw that caregivers with strong support networks are positively impacting the family unit. Often, these caregivers noted that they have access to taking respite for self-care like date nights with their partner; time away from the hospital to carry on their daily lifestyle; or accessing exercise like yoga, running, walking, or fitness. 

Lastly, the timing of mental health care in relation to where the PVS child is at in their treatment was discussed throughout our interviews. A new diagnosis or recurrent disease compared to a child with stable PVS may be a point of focus for mental health care for caregivers and their families. This can be explained by the length of time spent in the hospital and the acuity in care the caregiver witnesses. Caregivers note experiencing medical trauma related to prolonged hospitalizations, medical care, and the management of caring for a child with PVS.

“[…] we live that PTSD being in a locked unit, and you know, not being able to go when we want to, and running medications, and dealing with all of the side effects that come from the treatments and the medications, it’s extremely stressful […].”

As the child’s disease stabilizes, caregivers reported improvements in their symptoms of stress over time. This is seen in their mindset shifting from a survival mode towards a future-oriented outlook.

“Yeah, so now we are kind of at a stable spot. She from a day to day stand point, is living a normal life. She is eating by mouth now, we got to take her feeding tube out. She goes long stretches in between appointments. So it’s this, you feel normal, and then you go in for this appointment, you feel normal, and then you go in for a cath and there is all this news.”

Even with the improvements in their mental health over time, caregivers still express fear when going to appointments that their child’s disease will progress. This is important for understanding potential activators of the caregiver’s stress and coping and how it may impact the family unit. 

### 3.5. Mixed-Method Findings 

#### 3.5.1. SVI Was Not Associated with Parental Stress or Coping

In our quantitative analysis, neither stress nor coping was associated with social vulnerability. However, our sample was highly resourced, which may have buffered the effect of SVI. In our qualitative data, several parents acknowledged the importance of financial resources, including well-paying jobs, health insurance, and the ability of one parent to leave employment or work from home. Even caregivers who reported being financially stable expressed stress related to medical expenses. Additionally, caregivers who were providing health insurance through their work experienced stress related to maintaining health insurance during their child’s treatment. 

#### 3.5.2. Competing Family Demands and Role-Functioning-Related Stress

We found the number of additional children a caregiver had was positively associated with role functioning-related stress. In qualitative interviews, the majority of parents discussed balancing the needs of their children with PVS and their siblings. Caregivers report challenges balancing time and attention at the hospital and home. Throughout treatment, but particularly early on, caregivers felt a high demand to be present in the hospital with their child, necessitating leaving their siblings at home. To manage this added stressor, parents leaned on their support systems to carry out essential parenting responsibilities such as preparing meals, transporting children to school and activities, and completing house chores to maintain the home life for the healthy siblings while the caregivers are at the hospital.

#### 3.5.3. Disease Status and Communication-Related Stress

We also observed that parents of children with stable PVS had lower communication-related stress than those with children with newly diagnosed or recurrent PVS. In the qualitative interviews, caregivers discussed increased stress when communicating with their medical team and support network, particularly in the early stages of diagnosis and treatment. Caregivers described feeling confused and frustrated with their care team following the diagnosis due to a lack of clear communication and the limited information they had on PVS at this early stage. Communication issues also arose when the child with PVS experienced recurrent disease or received negative test results. Communication with the care team generally improved with the child’s health status. 

Caregivers frequently reported frustration with individuals in their support network who required repeated explanations from caregivers about PVS and the severity of their child’s illness. Particularly in the early stages, many caregivers dove into all available resources to learn about PVS. Although they did not expect their support network to do the same, caregivers were worn down by the repetitive task of teaching. Furthermore, PVS patients and their families each experience a unique disease pathway, which makes it difficult to describe their experience to others.

#### 3.5.4. Coping Patterns

Caregivers with more children reported lower family cohesion-related coping behaviors, highlighting the importance placed on strong family ties/difficulties balancing family needs while caring for a child with PVS; however, caregivers used diverse coping strategies. Balancing the needs of children with and without PVS often created tension. Caregivers often reported that the family would be separated during hospital stays, with either one parent traveling with the child with PVS, leaving their partner and other children at home, or both parents traveling with the child with PVS and leaving their other children with extended family. In response to this separation, caregivers prioritized family time when possible. 

In addition to relying on extended family to care for the siblings of children with PVS, the extended family also provided valuable respite for caregivers. Due to the medical complexity of caring for a child with PVS, traditional babysitters lacked the necessary skills. Conversely, the medical needs of some children with PVS did not rise to the level of a home health nurse. Thus, the extended family were often the only individuals able and willing to provide respite care. As time passed, caregivers found it difficult to maintain their support networks. Caregivers often reported feelings of guilt due to their resource needs remaining high. Caregivers also reported that their partners were important sources of comfort due to sharing the experience of having a child with PVS. 

Another commonly discussed coping resource was support groups. Many caregivers sought in-person or web-based support groups but expressed the need for greater access to PVS-specific support groups. Caregivers of children with PVS reported difficulty connecting with caregivers of children with CHD due to differences in the length of treatment and prognosis. Caregivers of children with PVS also described how socializing with other caregivers of children with PVS builds community and reduces isolation. 

## 4. Discussion

Our sample of caregivers of children with severe PVS described a high level of illness-related parental stress. Caregivers reported using multiple coping strategies and found social support the most helpful. Our primary predictor, SVI, was not associated with parenting stress or coping strategies. We observed that caregivers with more children reported higher role functioning-related stress, and caregivers of children with newly diagnosed or recurrent PVS had higher communication-related stress, although neither association was statistically significant. These associations, although not statistically significant, were consistent with the themes we observed in our qualitative analysis.

Caregivers of children with PVS reported higher levels of stress than parents of children with other serious illnesses of childhood, such as CHD. A study of caregivers of children with CHD reported an average difficulty PIP score of 73.6 out of 210 [8]. The parents in this study reported an average PIP score of 120, nearly 46 points higher, highlighting the significant stress related to caring for a child with PVS. Additionally, we found that caregivers of children with PVS reported higher stress at the time of diagnosis, which is consistent with caregivers of children with complex medical diseases like CHD [14]. Elevated stress following diagnosis was also reflected in our qualitative findings. This pattern is consistent among caregivers of children with CHD who report difficulty adjusting to their child’s disease and new parental role caring for a medically complex child [15]. 

Caregivers of children with PVS reported leaning on their partner for social support regardless of medical knowledge or role responsibilities. In a study of caregivers of individuals with CHD, family cohesion was the most helpful coping strategy [16]. A partner is uniquely situated to provide support based on proximity. The presence of a spousal partner has been shown to reduce anxiety and stress and improve a caregiver’s ability to cope [16,17,18,19]. 

The existing literature from the pediatric chronic illness community suggests that parenting stress increases when a family’s resources are limited [20]. Socioeconomic status is multifaceted, and studies have found varying associations between socioeconomic status and parental stress [21,22]. Common resources identified in previous studies of other chronic illnesses include access to reliable transportation to receive medical care, availability of social support for basic life needs, and ability to pay for medical expenses [23]. Caregivers from lower socioeconomic backgrounds face added social pressure when seeking financial assistance for their sick child and family [21]. Although participants in this study were relatively highly resourced, they still reported significant financial strain, emphasizing the high cost of caring for a child with a chronic condition. Within the rare disease community, larger systems of inequities exist, leading to less funding for research, patient organizations or foundations, policy initiatives, and other programs [24]. 

Prolonged hospitalizations and acute hospital care were significant stressors among caregivers of children with PVS and CHD [15,25]. Caregivers in both communities noted that waiting for medical results was highly distressing [26]. Similar to our findings, PTSD symptoms have also been observed in caregivers of children with CHD as a result of the high amount of stress they endure related to their child’s medical treatment [15,22,25]. Research on caregivers of children with CHD found that disease severity is associated with caregiver’s stress and mental health [15], which is consistent with our findings. 

Children with PVS need developmental care to be integrated into their medical care. As the treatment of PVS expands, more children with PVS will become hardware-limited and face multiple surgical interventions, reducing or restricting their time in traditional classrooms. PVS programs should support the development of children with PVS so they can actively participate and integrate into their community [5]. This is echoed in calls to action to build resources that will support children with PVS in thriving in school and engaging with their peers. 

### 4.1. Strengths and Limitations

This study has several limitations. The sample size was modest, which limited statistical power to detect significant associations. However, the sample was large for a study of caregivers of children with PVS, and we met thematic saturation. Our sample was also relatively high resourced, with an SVI score of 0.4, suggesting a low social vulnerability. This is likely due to inequitable access to comprehensive healthcare among children of lower socioeconomic status for this disease. Only one caregiver had a child who was newly diagnosed, likely due to stress impacting their ability to engage in our study. Lastly, we utilized a cross-sectional design that captured only one point in time. However, qualitative data and the inclusion of parents from multiple stages of treatment allow us to understand the changing needs within this population.

### 4.2. Recommendations

Caregivers recommended three key areas for improvement within healthcare facilities: education materials, expansion of the PVS NP role, and PVS support groups. A significant gap remaining in the PVS community is the lack of educational resources for caregivers and their families. Caregivers recommended the development and utilization of children’s books and materials for the child with PVS and their siblings, online resources and literature for caregivers, and preparatory materials on diagnosis and treatment. Caregivers express the importance of engaging with their care team in medical conversations. To address this, educational resources would support caregivers in entering these conversations more confidently. 

The role of the NP was discussed throughout our interviews, emphasizing the impact an NP has on establishing family-centered care. Caregivers stated that the NP explained medical information, reinforced quality of life measures, responded to financial stressors, and connected families to social and mental health supports. The role of the PVS NP is to provide continuity of medical care throughout the patient’s inpatient and outpatient treatment and support communication between families and their multidisciplinary care team. Healthcare systems should assess the requirements of the NP role and evaluate the capacity of the NP to respond efficiently and effectively to the needs of families. 

Access to formal and informal support groups and therapy was important to the family unit. Caregivers interviewed in this study wanted access to PVS-specific support groups, peer mentors, and therapy for the family unit throughout treatment. Several caregivers emphasized that these resources were needed early in treatment to provide support through this transition and build connections within the PVS community. 

Overall, caregivers note the benefits of a multidisciplinary approach to care. They highlight the importance of support through the continuity of providers. They express benefiting from access to psychosocial providers, including social workers, psychologists, child life specialists, and music therapists. They also acknowledged the importance of quaternary centers that have established PVS teams.

## 5. Conclusions

Our study found high levels of illness-related parental stress among caregivers of children with PVS. Stress evolved from what caregivers described as ‘survival mode’ to a future-oriented outlook. Caregivers indicated that communication and parental role functioning were coping strategies that could be better supported by providers and health systems. Currently, caregivers rely heavily on support networks that are not available to all caregivers or may experience strain over time. Therefore, we need support from the health system to promote equity. Building a community positively benefited the caregivers of children with PVS, and multiple caregivers identified a need to expand these support networks and systems to improve their health and well-being.

## Figures and Tables

**Table 1:** Child and caregiver characteristics.

Child Characteristic	N = 32
Age, median (IQR ^1^)	5.3 (6.3)
Female, Sex, N (%)	21.0 (65.6)
PVS status, N (%)	
Newly diagnosed	1.0 (3.1)
Recurrent	13.0 (40.6)
Stable	18.0 (56.3)
Diagnosed at BCH, N (%)	23.0 (71.9)
Distance from BCH, miles, median (IQR)	800.0 (1012.0)
Hospitalizations in past year, median (IQR)	3.0 (6.0)
Days hospitalized in past year, median (IQR)	2.0 (39.0)
**Caregiver Characteristic**	**N = 32**
Age, mean (SD ^2^)	39.0 (6.3)
Female, Sex, N (%)	29.0 (90.6)
Race/ethnicity, N (%)	
Asian	1.0 (3.1)
Black or African American	2.0 (6.3)
Hispanic	1.0 (3.1)
White	26.0 (81.3)
Black or African American/Native American	1.0 (3.1)
Preferred not to answer	1.0 (3.1)
Educational attainment, Master’s degree, N (%)	13.0 (40.6)
SVI ^3^, median (IQR)	0.4 (0.5)
Marital status, married/in a relationship, N (%)	29.0 (90.6)
Employment status, N (%)	
Full time	13.0 (40.6)
Part time	9.0 (28.1)
Unemployed or not working	10.0 (31.3)

^1^ IQR = interquartile range; ^2^ SD = standard deviation; ^3^ SVI = Social Vulnerability Index.

**Table 2:** Pediatric Inventory for Parents (PIP) Subscale Multivariable Regression results.

Variables	Communication Estimate (SE ^1^)	*p*-Value	Role Functioning Estimate (SE ^1^)	*p*-Value	Medical Care Estimate (SE ^1^)	*p*-Value	Emotional Distress Estimate (SE ^1^)	*p*-Value
SVI ^2^	1.9 (6.4)	0.77	1.0 (6.1)	0.87	−1.7 (8.0)	0.84	1.6 (11.9)	0.90
Caregiver Age, years	−0.04 (0.3)	0.89	0.2 (0.3)	0.49	0.3 (0.4)	0.52	0.3 (0.6)	0.60
Caregiver Sex, Female	5.5 (5.0)	0.28	5.5 (4.7)	0.26	5.3 (6.2)	0.41	14.6 (9.3)	0.13
Caregiver Relation, Partnered	−1.5 (6.0)	0.80	−4.3 (5.6)	0.45	1.5 (7.4)	0.84	−8.2 (11.1)	0.47
Race, White	0.3 (4.1)	0.93	−0.8 (3.8)	0.84	0.5 (5.1)	0.92	2.0 (7.5)	0.31
Children, N	0.7 (1.1)	0.55	1.9 (1.0)	0.08	1.1 (1.4)	0.44	2.2 (2.1)	0.30
Patient Age, years	0.0 (0.5)	0.96	−0.8 (0.5)	0.12	−0.5 (0.6)	0.40	−1.0 (1.0)	0.56
Patient condition, Recurrent/N.D. ^3^	5.8 (3.5)	0.11	3.8 (3.3)	0.26	3.6 (4.3)	0.42	3.8 (6.4)	0.79

^1^ Standard error; ^2^ SVI = Social Vulnerability Index; ^3^ N.D. = newly diagnosed.

**Table 3:** Coping Health Inventory for Parents (CHIP) Subscale Multivariable Regression results.

Variables	Family Cohesion Estimate (SE ^1^)	*p*-Value	Social Supports Estimate (SE ^1^)	*p*-Value	Communication Estimate (SE ^1^)	*p*-Value
SVI ^2^	2.3 (5.1)	0.66	3.3 (6.6)	0.62	3.6 (2.5)	0.17
Caregiver Age	0.1 (0.3)	0.66	0.1 (0.3)	0.67	−0.02 (0.1)	0.90
Caregiver Sex, Female	2.0 (3.1)	0.63	5.6 (5.2)	0.29	1.7 (2.0)	0.39
Caregiver Relation, Partnered	1.7 (4.8)	0.72	−0.4 (6.1)	0.96	−2.9 (2.4)	0.23
# of children	−1.4 (0.9)	0.13	−1.2 (1.1)	0.32	−0.6 (0.4)	0.19
Patient Age	−0.3 (0.4)	0.48	−0.2 (0.5)	0.70	−0.1 (0.2)	0.77
Patient condition, Stable	−1.3 (2.8)	0.64	−0.8 (3.6)	0.82	−1.1 (1.4)	0.43
Race, White	−1.4 (3.2)	0.67	−2.2 (4.2)	0.61	1.6 (1.6)	0.33

^1^ Standard error; ^2^ SVI = Social Vulnerability Index.

**Table 4:** Themes and sub-themes of qualitative interviews.

**Doman 1. Medical Diagnosis and Hospitalization**	
**Themes**	**Sub-themes**
Medical	Diagnosis, Treatment and Management, Hospitalization, Complications, Stabilization of disease, Rare disease, PVS Clinical Coordinator, Care Team
Hospital	Quaternary care, Familiarity with hospital, Hospital environment, Transfer, New city, Distance from hospital
Medical knowledge	Caring for child with PVS, Existing medical knowledge, Searching for information
**Domain 2. Family Unit**	
**Themes**	**Sub-themes**
Family	Partner, Family life, Siblings
Home	Home therapy, Medical routine, Child Care, School
Communication	Child with PVS, Clinical staff, Repeated Explanations
Finance	Donation/Gift, Work from home, Additional expenses, Stay at home, Health insurance
COVID	Isolation, Homeschool, Vaccination
**Domain 3. Mental Health and Coping**	
**Themes**	**Sub-themes**
Mental health	Sleep deprivation, Medical trauma, Anxiety/depression, Therapy, Burnout
Support system	Faith, Hospital Staff, Friends, Informal support group, Family, Facebook, Formal support group
Self-care	Hygiene, Writing/blogging, Outdoors, Breaks, Exercise
Coping	Leaving the bedside, Humor, sarcasm. Changing needs, Self-awareness, Looking to the future, Living in the moment, Giving back

## Data Availability

The original data presented in the study are not available due to the privacy of personal information and rarity in the disease population.

## Supplementary Materials

The following supporting information can be downloaded at: https://www.mdpi.com/article/10.3390/children11081008/s1, Table S1. Qualitative Interview Guiding Questions, Table S2. Coping Health Inventory Results, and Table S3. Pediatric Inventory for Parents Results.
