# Supplementary material for: Exploring Stress and Coping in Caregivers of Children with Pulmonary Vein Stenosis: A Mixed-Method Study
Source: Children (Basel). 2024 Aug 17;11(8):1008. doi: 10.3390/children11081008 (PMC11352767; doi:10.3390/children11081008)
Supplement: Supplementary file 1 [file children-11-01008-s001.zip › children-3129152-supplementary.pdf]

Supplemental Material

Table S1. Qualitative Interview Guiding Questions

Guiding Questions
1. Tell me about your experience having a child with PVS, starting with the time your child received their diagnosis?
2. How would you describe the impact of your child's diagnosis on your family?
3. What kinds of coping strategies such as tools or practices have you used to manage your stress?
4. Can you tell me what your support system looks like now having a child with PVS?

Table S2. Pediatric Inventory for Parents, (PIP) Results

Stressor	Frequency Mean (SD)	Difficulty Mean (SD)
Communication [1]	23.0 (7.4)	20.7 (7.1)
Role Functioning [2]	27.0 (7.7)	27.3 (7.8)
Medical Care [3]	25.1 (8.7)	20.5 (7.4)
Emotional Distress [4]	45.2 (13.8)	48.4 (13.0)

[1] Max score 45, [2] Max score 50, [3] Max score 40, [4] Max score 75

Table S3. Coping Health Inventory, (CHIP) Results

Coping Pattern	Mean (SD)
Social Supports [1]	37.2 (7.2)
Communication [2]	15.6 (3.0)
Family Cohesion [3]	25.8 (5.6)

[1] Max score 54, [2] Max score 24, [3] Max score 48
